# Exploratory Assessment of Health-Related Parameters in World-Class Boccia Players Using DXA

**DOI:** 10.3390/healthcare13141658

**Published:** 2025-07-09

**Authors:** Bárbara Vasconcelos, José Irineu Gorla, Karina Santos Guedes de Sá, Rui Corredeira, Tânia Bastos

**Affiliations:** 1 Centre of Research, Education, Innovation and Intervention in Sport (CIFI2D), Faculty of Sport, University of Porto, 4099-002 Porto, Portugal; fontes.b94@gmail.com; 2 Department of Adapted Physical Activity Studies, Faculty of Physical Education, State University of Campinas, Barão Geraldo 13083-859, Brazil; jigorla@uol.com.br (J.I.G.); karina-sa@outlook.com (K.S.G.d.S.); 3 ACELERAR Development Center, Rolândia 86605-000, Brazil; 4 Research Center in Physical Activity, Health and Leisure (CIAFEL), Faculty of Sport, University of Porto, 4099-002 Porto, Portugal; rcorredeira@fade.up.pt; 5 Laboratory for Integrative and Translational Research in Population Health (ITR), 4050-600 Porto, Portugal

**Keywords:** Paralympic sports, cerebral palsy, body composition, obesity, sarcopenia risk, bone health, osteoporosis, diagnostic imaging

## Abstract

**Background**: Sport plays an important role in the health promotion of people with cerebral palsy (CP). However, risk factors may impair sport performance and health in non-ambulatory athletes. Therefore, the aim of the present study was to explore body composition and bone health in a group of world-class Boccia players with CP. **Methods**: Five BC2-class players with CP, aged 15–42 years old, were assessed using Dual-Energy X-Ray Absorptiometry (DXA) for body composition and bone mineral density (BMD) and content (BMC). The fat mass index (kg/m^2^) was used to define obesity, and the BMD Z-score used to analyze bone health. A preliminary indicator of sarcopenia was considered using the appendicular lean mass index. **Results**: Players 1 and 3 exhibited similar body compositions (obesity class 1 and BMD Z-score are below the expected range for age). Player 5 exhibited multiple health-related risk factors. The results regarding youth players (Player 2 and Player 4) should be analyzed with caution. **Conclusions**: Overall, due to Boccia’s specific characteristics, players may benefit from close monitoring by multidisciplinary teams and supplementary strategies (e.g., strength training, individualized diet plans) to promote quality of life and performance. However, further research is needed to confirm the data, since these preliminary findings do not allow for broader generalizations.

## 1. Introduction

Cerebral palsy (CP) is a clinical description for a set of permanent developmental disorders affecting posture and movement, leading to activity limitations. The global prevalence has remained relatively consistent over the last decades, affecting approximately 2 to 3 out of every 1000 live births [1]. CP is a non-progressive injury that occurs in the developing brain of a fetus or child. It is typically accompanied by sensory, cognitive, communication, perception, and behavioral disorders, as well as epilepsy and secondary musculoskeletal issues [2]. CP types can be categorized into three primary syndromes: spastic, dyskinetic, and motor ataxia [3]. Specifically, spastic CP, resulting from cortical damage, is the most prevalent form of CP and is characterized by spasticity, hyperreflexia, and slow and effortful voluntary movements [4]. In general, people with CP exhibit a wide range of clinical symptoms and are described as a heterogeneous group.

Participation in sports has been identified as a key factor influencing overall health and quality of life for people with CP. It is crucial that people with CP engage in sports or any physical activity as a health-promoting measure. In this sense, Groff, Lundberg [5] emphasizes the importance of inclusive practices in para sports to support the well-being and social inclusion of this population. Specifically, Boccia is a Paralympic sport originally developed for people with CP [6] and is described as a highly strategical and precision game that consists of placing red or blue balls as close to the target (white ball) as possible [7]. Even though it is currently being played by athletes with different physical disabilities (e.g., spinal cord injuries or muscular dystrophy), CP players with severe quadriplegia represent the majority of the Boccia players [8]. Boccia improves life satisfaction, transforms exercise into an enjoyable activity, and gives players a sense of purpose and self-worth [9]. However, many people with CP report a diminished quality of life due to limited inclusion in health and community activities [10].

As people with CP transition into adulthood, they often face significant secondary health conditions due to physical inactivity, which leads to decreased physical fitness levels. This lack of physical activity not only reduces overall functional capacity but also raises the risk of obesity, further complicating health outcomes for adults with CP [11]. Overall, people with CP are at risk for different health parameters due to the neurological disorder itself, physically inactive lifestyles, medication, and lack of mobility when compared with the general population.

Specifically, one of the most concerning health issues is the extreme variation in body composition among people with CP. People with physical impairments, including CP, usually present differences in body composition patterns that may be characterized by increased fat mass or loss of lean mass [12,13]. Moreover, people with tetraplegia develop an increased deposition of subcutaneous and visceral fat and a reduction of lean mass in the upper and lower limbs [14]. This situation is more evident in people with severe forms of CP. For example, Shin and Jung [11] found that an ambulatory group of adults with CP had more people with a normal fat mass index (FMI), while a non-ambulatory group had more people considered fat-deficient and obese. Therefore, more research is needed to characterize body composition variables in people with CP, specifically those engaged in a sports setting.

It is important to highlight that, even though there are no studies analyzing the relation between body composition and performance of Boccia players, in other Paralympic sports, such as para swimming [15,16] and track and field [17], this analysis was already conducted. Medeiros, Alves [15] observed a significant relationship between reduced fat mass percentages and improved performance among Brazilian swimmers after six months of training. Additionally, Dingley, Pyne [16] emphasized that specific physical attributes, such as lower skinfold thickness, contributed positively to swimming propulsion and efficiency. On the other hand, Cherif, Said [17] demonstrated a negative correlation between physical performance (e.g., performance of the drop jump, repeated sprints ability) and body composition measures among track and field athletes with CP. Overall, it is suggested that physical fitness and, consequently, sport performance are affected by body composition in athletes with CP.

A conceptual model about the cyclical nature of health and functional decline of people with CP identifies muscle weakness and fatigue as the main features leading to the development of premature sarcopenia [18]. Sarcopenia is a progressive skeletal muscle disease characterized by reduced muscle quantity or quality, and it is diagnosed when at least two of the following criteria are met: reduced muscle strength, diminished muscle quality or quantity, and/or compromised physical performance [19]. According to Su, Tsai [20], the previously mentioned criteria often occur simultaneously in people with CP, with adults presenting a higher prevalence of sarcopenia when compared to the general population [21]. Consequently, sarcopenia heightens cardiometabolic risk and accelerates the functional decline of people with CP [18]. However, to the best of our knowledge, no previous studies characterized sarcopenia risk among players with CP.

Another important health-related parameter in people with CP is bone health. People with neurological disorders that cause quadriplegia face a significant risk of developing osteopenia or osteoporosis due to reduced bone density [22]. Won and Jung [23] reported that decreased BMD was associated with male sex, age, decreased motor function, loss of ambulatory function, and low BMI. Specifically in young adults with CP, ambulatory status [24], type of CP [25], and Gross Motor Function Classification System (GMFCS) scores [26] were associated with BMD. Also, relatively low bone mass in the lumbar spine, hip, and femur was identified in this group [22]. Overall, low BMD represents a common health issue in people with CP, regardless of age, which leads to a high prevalence of fractures [27]. People with CP have an annual fracture rate of around 5%, which is twice as high when compared with the age-matched general population [20]. Therefore, it is crucial to characterize bone mass in athletes with CP due to the impact that training and competition loads can have on athletes’ bone health.

Overall, underweight or obesity, sarcopenia, and osteoporosis comprise a triad of health issues that may affect people with CP. From a health perspective, physical activity and sport may have an important preventive contribution to the above-described triad of comorbidities and their interactions throughout the lifespan of people with CP. However, there are limited insights into critical aspects of the body composition and bone health in athletes with physical disabilities [28], specifically with CP. Consequently, a better understanding of body composition of world-class Boccia players is needed not only for sport performance requirements but also for athletes’ long-term health.

Therefore, this study aimed to explore body composition and bone health, including fat mass, lean mass, and BMD, among a group of world-class Boccia players with CP using DXA. Additionally, a correlation analysis was performed among sociodemographic, body composition, and bone health variables. Preliminary health-related risk indicators, such as underweight or obesity, preliminary sarcopenia risk, and osteoporosis, were identified, and strategies to optimize sport participation and health maintenance in players with CP were suggested.

## 2. Materials and Methods

### 2.1. Participants

A total of five BC2 Boccia players, aged between 15 and 42 years old and diagnosed with CP, were recruited to participate in the present study. The inclusion criteria encompassed the following: (i) a confirmed diagnosis of CP, regardless of its subtype; (ii) classification as BC2; (iii) participation in the national Boccia team during the preceding season; and (iv) participation in training sessions during the current season. The exclusion criterion was related to participants who had never received a call-up to the national Boccia team. The BC2 class was chosen because it represents the sport class with a greater number of players with CP in the Portuguese national team. All participants were European or world medalists. Regarding locomotion type, all were non-ambulatory and used electric wheelchairs.

Table 1 presents an overview of the participants’ personal and clinical-related characteristics, including gender, type of CP, and presence of associated diseases.

### 2.2. Procedures

Data collection took place in the Research Centre for Physical Activity, Health, and Leisure (CIAFEL) laboratory located at the Faculty of Sport, University of Porto. All participants received a comprehensive verbal explanation of the protocol details. Upon voluntarily agreeing to participate, the participants signed a consent form containing a written explanation of the protocol. Informed consent was obtained from the underage participants’ tutors, as well as from the participants themselves. The research protocol received approval from the faculty Ethical Committee under the reference number CEFADE 47/2022.

### 2.3. Instruments

The participants filled out a sociodemographic questionnaire including personal information, such as age, gender, height (m), weight (kg), and sports-related information, such as years of training and competition, training frequency per week, and duration (min) per session for both specific and strength and conditioning (S&C) training. Additionally, clinical information was collected, including the type of CP, walking capability, type of walking assistive device used, and associated diseases. Nutritional monitoring was also inquired about.

A Dual-Energy X-Ray Scan (Explorer QDR 4500, Hologic, Bedford, MA, USA) was used to assess the total fat mass (FM), total body% fat, total lean mass, and appendicular lean mass. Also, to assess BMD and BMC, whole-body, radius, and lumbar spine scans were used.

The body mass index (BMI) was calculated as body mass (kg) divided by height squared (m^2^). The fat mass index (FMI) was calculated as FM (kg) divided by height squared (m^2^). The FMI categories were defined as follows: severe fat deficit in men with <2 kg/m^2^ and women with <3.5 kg/m^2^; moderate fat deficit in men with 2 kg/m^2^ to less than 2.3 kg/m^2^ and women with 3.5 kg/m^2^ to less than 4 kg/m^2^; mild fat deficit in men with 2.3 kg/m^2^ to less than 3 kg/m^2^ and women with 4 kg/m^2^ to less than 5 kg/m^2^; normal fat levels in men with 3 kg/m^2^ to 6 kg/m^2^ and women with 5 kg/m^2^ to 9 kg/m^2^; excess fat levels in men with more than 6 kg/m^2^ up to 9 kg/m^2^ and women with more than 9 kg/m^2^ up to 13 kg/m^2^; obesity class 1 in men with more than 9 kg/m^2^ up to 12 kg/m^2^ and women with more than 13 kg/m^2^ up to 17 kg/m^2^; obesity class 2 in men with more than 12 kg/m^2^ up to 15 kg/m^2^ and women with more than 17 kg/m^2^ up to 21 kg/m^2^; and obesity class 3 in men with more than 15 kg/m^2^ and women with more than 21 kg/m^2^ [29]. The total body %FM was calculated as FM divided by the total body mass (kg). The appendicular lean mass index (kg/m^2^) was calculated as appendicular lean mass (kg) divided by height squared (m^2^) and used as a preliminary indicator of sarcopenia risk. The cut-off points according to gender were <7.0 kg/m^2^ (men) and <5.5 kg/m^2^ (women) [19].

BMD (g/cm^2^) and osteoporosis were defined using the Binkley, Bilezikian [30] cut-off points, where Z-scores (age, gender, and ethnicity-matched) are used for females prior to menopause and males younger than the age of 50. A Z-score of −2.0 or lower is defined as “below the expected range for age”, while a Z-score above −2.0 is considered “within the expected range for age”.

Regarding the DXA measurement procedures, the same trained operator performed all scans and analyses to ensure consistency. The participants were placed in a supine position with arms in extension near the trunk and lower limbs in extension, with a slight abduction of the feet. Some participants needed foam pads to enable consistent positioning and separation of the limbs. The foam was used because it is not detectable by the DXA scanner. Due to difficulties with positioning on the DXA and post-analysis of the scans, only full-body values were used for body composition. The participants’ clothing was kept to a minimum, and all metallic objects (e.g., earrings, watches) as well as shoes were removed.

### 2.4. Statistical Analysis

The descriptive statistics, including mean, standard deviation, and minimum and maximum values, were calculated. A Shapiro–Wilk test, confirming the normality of the data, was performed. Pearson correlations were conducted between all variables, and the coefficient of determination (r^2^) was reported. An r^2^ close to 0 indicates a weak relationship; values between 0.3 and 0.5 suggest a moderate relationship, while those above 0.7 indicate a strong explanatory model. An r^2^ of 1 means a perfect fit [31]. For all analyses, the significance level adopted was a *p*-value ≤ 0.05. The statistical package R [32] was used to carry out the analyses.

## 3. Results

Table 2 presents a detailed description of sociodemographic variables, body composition, BMD, and BMC parameters for whole-body, radius, and lumbar spine regions.

In relation to the sociodemographic parameters, the participants included both female (n = 2) and male (n = 3) players, as well as adults (n = 3) and young players (n = 2). There was a wide interval of sport experience, with years of Boccia practice ranging from 3 to 25 years and years of Boccia competition ranging from 2 to 25 years. The frequency of Boccia training ranged from two to six sessions per week (including specific and S&C training).

Specifically, Player 1 (male, 42 years old) was the oldest participant and reported the highest volume of training per week (duration × frequency of specific training per week). He was classified as obesity class I [29] and exhibited the highest ALM and ALMI. The BMD Z-score was below the expected range for age.

Player 3 (male, 41 years old) reported the highest number of years of practice and competition as well as the highest volume of S&C training (duration × frequency of S&C training per week). Player 3 also exhibited obesity class I, a preliminary indicator of sarcopenia risk, and the BMD Z-score was below the expected range for the age.

Player 5 (female, 31 years old) reported the longest S&C training duration. The total fat mass and FMI scores were the highest among all the players and were classified as obesity class I [29]. Regarding bone health, she also exhibited the lowest BMD Z-scores.

Among the youth group, both Player 2 (male, 15 years old) and Player 4 (female, 16 years old) reported an adequate training volume considering the age. Both exhibited normal fat levels and lower ALM and ALMI scores when compared with the adult cut-off values. Regarding bone health, the BMD z-scores were considered below the expected range for the age. However, the scores need to be interpreted with caution due to the young age of the players and considering their developmental stage.

With the exception of Player 5, all players reported nutritional monitoring offered by the club or primary health care center. None of the players reported supplementation (e.g., calcium, vitamin D) or medication intake known to affect bone metabolism.

A correlation analysis was performed among all sociodemographic, body composition, and bone health variables. The significant correlations are reported in Table 3. Overall, strong and positive associations were identified among the variables.

## 4. Discussion

This study aimed to explore body composition and bone health among a group of world-class Boccia players with CP using DXA. Specifically, health-related risk factors were identified, and strategies to optimize sport performance and health maintenance in players with CP were suggested.

Despite the relevance of Boccia in the context of sport for persons with disabilities, there is a scarcity of studies that provide scientific-based evidence about this Paralympic sport. In accordance, Ferreira, Espada [33] developed an up-to-date systematic review about the status of Boccia research. The previously mentioned authors only identified nine studies and concluded that Boccia has been consistently neglected in research when compared with other Paralympic sports. Moreover, Boccia players, including those with severe CP, are part of a minority group with great inter-individual variability, difficult access to the study participants [6], and important methodological limitations when accessing non-ambulatory athletes. Overall, this may explain the lack of opportunity and interest of the scientific community in studying Boccia players.

To the best of our knowledge, this was the first study to use DXA in Boccia world-class players with CP to assess body composition. DXA is a sophisticated method to assess body composition and bone health with several advantages for research because it is a fast, accurate, and easy-to-use methodology that allows for regional measurements and less radiation when compared with Computed Tomography scans, chest, and lumbar spine X-rays, although it is not recommended for regular assessments [34]. Moreover, the use of DXA allows for overcoming the limitations of BMI assessments. BMI is a frequently used method, but it is not accurate to assess overall adiposity for adults with CP given the diversity of body morphology and lifestyles [11]. However, in the present study, the use of DXA in players with CP also raised several methodological issues related to body alignment and spasticity that reduced the number of reliable data available and restricted the understanding about asymmetries in this group of non-ambulatory players. Therefore, it is crucial to develop tailored and specific methods to assess the body composition of people with CP. The use of portable DXA technology, combined with protocols designed to address the unique challenges of players with severe CP, could enhance the accuracy and reliability of the data. For example, specific protocols could include customized positioning aids and adjustments to account for postural asymmetries, allowing for the successful evaluation of players with spasticity or movement limitations.

Lastly, to the best of our knowledge, this was the first study to explore the importance of high-performance Boccia in promoting health-related parameters. While the latest research in the field only focuses on Boccia sports’ performance factors [33,35,36,37], the assessment of health-related parameters enhances the need for health monitoring in an understudied population of Paralympic athletes [38].

Regarding the sport variables, all players reported an extensive volume of training per week, including technical and tactical Boccia training and S&C training. Considering that Player 1 (male, 42 years old), Player 3 (male, 41 years old), and Player 5 (female, 31 years old) were classified as obesity class 1, it appears that this volume is not sufficient or adequate for these players. However, it is important to highlight that normative or specific cut-off values to analyze the body composition of people with CP are not available in the literature. Therefore, in the present study, normative values used for the general population were applied, which may limit the interpretation of the findings. Although a common approach in previously published studies within the same scope, we cannot exclude that the use of general normative values can lead to an overestimation or underestimation leading to a misclassification of players with CP.

Similarly, Cavedon, Sandri [28] assessed the body composition of SCI athletes competing in different Paralympic sports (e.g., handbike, wheelchair rugby, and wheelchair basketball) and reported that 58.3% were classified as obese. McPhee, Claridge [39] argued that obesity is an important risk factor for a higher prevalence of cardiovascular disease (CVD) in people with CP. Adults with severe CP present several risk factors, such as obesity and overweight, hypertension, low-density lipoprotein cholesterol, and a higher risk of atherosclerosis due to the increase in thickness of the carotid artery intima media [18,39]. Consequently, there is an increase in CVD-related mortality [40]. Although it is difficult to determine if adults with CP face a higher prevalence of overweight or obesity when compared to the general population [39], the findings of the present study suggest a similar unfavorable trend among these groups of world-class players with CP. Therefore, monitoring and managing common risk factors is essential to prevent the onset of CVD in adults with CP [39]. Gorla, Costa e Silva [41] assessed the longitudinal effects of wheelchair rugby (WR) training on the body composition of players with tetraplegia and found that WR training increases the lean mass in the arms and decreases the FM in the trunk and legs, demonstrating that WR can have a positive effect on the weight management of the players. Similarly to WR, Boccia primarily involves upper-body movements to perform [35], but both sports are very different in terms of intensity of the effort and, consequently, cardiovascular capacity performance. Boccia training requires limited aerobic or anaerobic intensity and may not generate enough metabolic demand to efficiently manage adiposity or fat mass.

In this sense, Boccia S&C training as a strategy for Player 1 (male, 42 years old), Player 3 (male, 41 years old), and Player 5 (female, 31 years old) could include two to four sessions per week lasting 20 to 60 min of steady and rhythmic moderate or vigorous physical activity, engaging major muscle groups [42]. Also, strength training could be conducted 2–3 times per week with 2–4 sets of 6–12 repetitions before failure [42]. S&C training is also important to improve aerobic capacity and increase energy reserves [43], crucial to performing a full schedule in a world-class competition. For example, at the Paris 2024 Paralympic Games [44], a male BC2 player reaching the final in the individual competition played a total of seven games over a four-day period. Additionally, participation in the BC1/BC2 team events would add five games over three days of the competition. This demanding schedule requires energy reserves and aerobic capacity to sustain high performance levels across multiple games, recover between games, and minimize the risk of fatigue-related errors that may compromise the player’s ability to compete successfully. All of these training strategies would contribute to managing weight and ensure a proper cardiovascular capacity for health purposes.

In addition to training strategies, all players would benefit from proper individualized diet plans with specific food recommendations prescribed by a specialized nutritionist. Also, a regular anthropometric assessment would help monitor body composition improvements and adjust the previous prescribed diet plan [45]. Specifically, Player 5 (female, 31 years old) would benefit from nutritional monitoring in terms of health and performance, considering the absence of intervention in this area. Also, it is important to explore the use of optical heart rate monitors to quantify the exercise intensity during training sessions and provide a better adjustment of the diet plan to the needs of the players. Therefore, future studies could characterize the energy expenditure in Boccia, which has already been performed in other para sports [46].

Player 5, the only adult female in the study (31 years old), presented a preliminary indicator of sarcopenia risk due to low ALMI. For this specific player, strength training is crucial to prevent muscle loss. For example, exercises could progress from primarily single-joint, machine-based resistance exercises to a combination of machine and free-weight, multi-joint, closed-kinetic chain exercises [42]. In addition to the strategies previously provided about FITT principles (frequency, intensity, time, and type), muscular hypertrophy could be added using loads of 60–80% of the one-maximum repetition of the player [42]. Strength training is of crucial importance for Boccia players due to performance factors related to the ability to throw at a longer distance [8] but also to support muscle development and minimize the risk of sarcopenia, emphasizing the importance of exercise and physical activity to improve muscle health in people with CP [42]. Stretching and mobility training could also be included to maintain postural control [47].

Moreover, regarding supplementation strategies suggested by the nutrition practitioner, and considering the recommendations of the American College of Sports and Medicine for protein intake between 1.2 and 2.0 g/kg/day in athletes [48], Player 5 (female, 31 years old) could benefit from increases in lean body mass [49], with proper protein ingestion. However, due to the lack of evidence about protein intake for Boccia players with CP, further research is needed. Also, creatine supplementation appears to be effective in muscle growth, but a lack of research with muscular disease-related populations enables clear recommendations for players with CP [50].

Finally, regarding bone health parameters, the present study revealed low BMD and BMC in Player 1 (male, 42 years old) and Player 5 (female, 31 years old). A similar outcome was also reported in studies with non-athletes with CP. For example, Won and Jung [23] assessed BMD in adults with CP and reported that 25% had osteoporosis, with a prevalence rising to 65% in persons over 50 years old. Moreover, men, non-ambulatory and those with bilateral CP, were at a higher risk for lower BMD.

When analyzing the previous literature about the assessment of BMD in athletes with disabilities, Koivisto-Mork, Steffen [51] also found that 50% of Norwegian Paralympic athletes had low BMD, with 29% exhibiting osteoporotic BMD levels and non-ambulatory athletes with congenital conditions being at a higher risk for low BMD when compared to other para-athletes. Although the previously mentioned study encompassed elite athletes from different sports and types of disabilities, Boccia was not included, and only five ambulatory athletes with CP were assessed. Ambulatory status is an important factor when analyzing the contribution of sports in preventing osteoporosis in athletes with disabilities. Miyahara, Wang [52] explored the effects of sports activity and found that wheelchair-dependent athletes had significantly lower BMD Z-scores in the whole body than non-wheelchair-dependent athletes. Similarly, Cavedon, Sandri [28] reported significantly lower BMD in athletes with SCI, with 45.8% reporting osteopenia and 12.5% osteoporosis. However, the two previously mentioned studies only included athletes with SCI. In the only study found with Boccia players, Weijer, van Dijk [53] also concluded that wheelchair-dependent athletes exhibited significantly lower BMD Z-scores and a higher prevalence of osteoporosis when compared to non-wheelchair-dependent athletes at the whole-body level. Mechanical loading patterns, including wheelchair use, the type of sport, and body mass, were identified as the primary risk factors. However, a study by Weijer, van Dijk [53] only included one Boccia player, and specific information about this participant was not available.

In addition, considering that reduced BMD is associated with an increased risk of fractures, even under minimal stress, negatively impacting the quality of life [23], it is important to identify potential trauma situations, such as transfers or handling gym equipment.

Therefore, it is possible to conclude that the Boccia players assessed in the present study may present vulnerable bone health, and complementary nutritional support may play a pivotal role. For example, vitamin D status and dietary intake of calcium could be monitored in blood analysis due to their importance regarding bone density. Some players may need complementary strategies, such as oral nutritional supplements in the form of milk- or fruit-based drinks or vitamin and mineral supplements prescribed by the multidisciplinary team, in compliance with the anti-doping code [54]. Moreover, because Boccia is an indoor sport, all players could take specific supplements to avoid deficiency of vitamin D [55].

Considering all of the abovementioned health variables, Player 5 (female, 31 years old) presented a vulnerable health status, exhibiting multiple health-related risk factors, namely obesity class 1, a preliminary indicator of sarcopenia risk, and bone mass below the expected range for age. It is also important to highlight that Player 5 was the only participant without nutritional monitoring. The present findings suggested very poor health status, with a possible impact on the player’s life expectancy, quality of life, and ability to maintain high-level Boccia performance over time.

Specifically, considering Player 2 (male, 15 years old) and Player 4 (female, 16 years old), who represent the youth players of this study, both were classified as normal fat and exhibited low ALMI. ALMI scores have to be interpreted with caution due to the young age of the players. In youth, low ALMI reflects developmental factors rather than pathological loss, hindering conclusive muscle atrophy claims. Also, the BMD z-scores were considered below the expected range for age for both players. Therefore, health strategies could include skeletal loading and nutritional strategies appropriate to this developmental stage.

Finally, the associations between different variables confirmed the findings previously stated, with the exception of the correlation between age and BMC. Regarding sociodemographic and body composition variables, the associations suggested that older players tend to have a higher weight, and greater weight was associated with an increased fat mass. In terms of bone health, the findings indicated that players with higher BMD also exhibited greater BMC. However, older age was associated with higher BMC, which is not an expected result, considering previous research on people without disabilities. According to Kelly, Wilson [29], BMC typically declines with aging in the general population. Therefore, more research is needed to clarify relationships and interactions between different health-related variables in the population with CP.

## 5. Limitations and Suggestions for Future Research

The present findings should be interpreted with caution due to some methodological considerations. Firstly, the small sample size limits the ability to draw broad conclusions about Boccia players with CP. Expanding the sample would allow comparisons between genders, age groups, and other variables with impact on body composition and bone health measures. However, gathering a representative sample of Boccia players with CP is very challenging due to its reduced number as well as the severity of the disability and complexity of daily life activities. For example, the majority of the players rely on others to ensure transportation and functional mobility for daily living. These daily life challenges also hindered standardization of DXA scans in some protocol procedures (e.g., time of day). To overcome these barriers, the establishment of international collaborations could improve the studies’ representativeness. Partnerships between Boccia clubs and national teams within the World Boccia organization could facilitate multi-site research aggregating data from different countries.

Secondly, future studies aiming to diagnose sarcopenia in players with CP should include the full assessment criteria for reduced muscle strength, diminished muscle quality or quantity, and/or compromised physical performance.

Thirdly, the lack of specific information about the nutritional status of Boccia players is also a limitation. Nutrition plays a crucial role in managing weight and reducing the risk of obesity in contributing to hypertrophy by combining specific S&C programs with proper protein and carb ingestion and in reducing the risk of osteoporosis by providing adequate vitamin D and calcium intake. Considering that the majority of participants were categorized as obesity class I, future studies should offer a behavioral lifestyle intervention that combines structured S&C Boccia programs with an individualized healthy diet. A practical intervention could include nutrition education programs (e.g., workshops) developed by sports nutritionists specifically for Boccia players, sports assistants, and families. This would provide evidence-based recommendations for balanced nutrition to enhance athletic performance and overall health in people with CP.

Lastly, the height and weight of the players were self-reported, which could have implied measurement error. Future studies should use objective measures and proper equipment for non-ambulatory players.

Despite the current limitations, the preliminary findings could help understand the importance of body composition and bone health on the long-term health of Boccia players with CP as well as their ability to keep competing at a world-class level over time.

## 6. Conclusions

The results from this exploratory study with a group of world-class Boccia players with CP revealed that specific characteristics of the Boccia game may limit the physiological health benefits commonly associated with other Paralympic sports. Consequently, high-performance Boccia training is unlikely to counteract preliminary health risks such as obesity, sarcopenia, or osteoporosis, emphasizing the need for supplementary S&C programs and individualized nutritional strategies. A first step toward health could be integrating health-promoting strategies into Boccia training, regardless of the competitive level. Such efforts can optimize sport performance, enhance quality of life, and highlight the important role of Paralympic sports in promoting health and inclusion for people with CP.

## Figures and Tables

**Table 1:** Personal and clinical characterization of the participants.

Variable	n (%)
**Gender**	
F	2 (40)
M	3 (60)
**Type of CP**	
Spastic quadriplegia	5 (100)
**Associated diseases**	
No	4 (80)
Arrhythmia	1 (20)

Note: F = female; M = male; CP = cerebral palsy.

**Table 2:** Sociodemographic variables, body composition, BMD, and BMC parameters for the whole-body, radius, and lumbar spine regions of the players.

			Mean	SD	Player 1	Player 2	Player 3	Player 4	Player 5
Sex			-	-	Male	Male	Male	Female	Female
Age			29	13.06	42	15	41	16	31
Height (cm)			157.4	10.06	152.0	165.0	170.0	145.0	155.0
Weight (kg)			58.4	19.53	75.0	42.0	74.0	33.0	68.0
Years of practice			13.80	8.11	15	10	25	3	16
Years of competition			11.20	9.58	10	3	25	2	16
Specific training sessions: duration per session (min)			183.00	65.73	300	150	150	150	165 *
Specific training sessions: frequency per week			4.40	1.14	5	4	6	3	4
S&C training sessions: duration per session (min)			43.00	14.40	20	45 *	45	45	60
S&C training sessions: frequency per week			3.25	1.50	5	2	4	2	2.5 *
Nutritional monitoring			-	-	Yes	Yes	Yes	Yes	No
BMI (kg/m^2^)			23.50	7.65	32.5	15.4	25.6	15.7	28.3
Whole body		Fat mass (kg)	22.31	11.17	27.169	9.598	29.874	11.127	33.798
		FMI (kg/m^2^)	9.00	4.44	11.76	3.52	10.34	5.29	14.07
		% Fat	37.82	10.63	38.60	22.50	42.90	34.00	51.10
		Lean Mass (kg)	32.53	7.98	41.398	31.897	37.844	20.484	31.016
		ALM (kg)	12.80	3.87	16.63	12.80	16.21	7.27	11.08
		ALMI (kg/m^2^)	5.12	1.39	7.20	4.70	5.61	3.46	4.61
		BMD (g/cm^2^)	0.941	0.065	0.966	0.907	1.036	0.865	0.929
		BMD Z−score	−2.1	0.3	−2.4	−2.0	−1.6	−2.4	−2.3
		BMC (g)	1463.27	345.27	1811.21	1225.16	1858.47	1123.09	1298.43
Radius	1/3	BMC (g)	1.57	0.31	2.05	1.32	1.7	1.43	1.35
		BMD (g/cm^2^)	0.66	0.07	0.763	0.636	0.713	0.601	0.599
		BMD Z−score	−1.30	0.35	−0.8	−1.1	−1.7	−1.5	−1.4
Lumbar Spine		BMC (g)	44.31	12.69	40.54	50.1	63.03	30.03	37.83
		BMD (g/cm^2^)	0.85	0.16	0.779	0.906	1.089	0.672	0.818
		BMD Z−score	−1.70	1.50	−2.7	−0.4	0.1	−3.4	−2.1

Note: SD = standard deviation; S&C = strength and conditioning; BMI = body mass index; FMI = fat mass index; ALM = appendicular lean mass; ALMI = appendicular lean mass index; BMD = bone mineral density; BMC = bone mineral content; * mean of the reported intervals.

**Table 3:** Correlation analysis among the different groups of variables.

Variables	R	*p* Value	r^2^
Weight × Age	0.96	0.009	0.93
Weight × Fat mass	0.93	0.028	0.84
Age × BMC	0.93	0.024	0.86
BMD × BMC	0.92	0.027	0.85

Note: BMD = bone mineral density; BMC = bone mineral content; R = correlation coefficient; r^2^ = coefficient of determination.

## Data Availability

Data are contained within the article.
